# The Scientific Self: Reclaiming Its Place in the History of Research Ethics

**DOI:** 10.1007/s11948-017-9945-8

**Published:** 2017-07-18

**Authors:** Herman Paul

**Affiliations:** Institute for History, Leiden University, P.O. Box 9515, 2300 RA Leiden, The Netherlands

**Keywords:** Research ethics, Scientific self, History of ethics, Moral economies, Epistemic virtues

## Abstract

How can the history of research ethics be expanded beyond the standard narrative of codification—a story that does not reach back beyond World War II—without becoming so broad as to lose all distinctiveness? This article proposes a history of research ethics focused on the “scientific self,” that is, the role-specific identity of scientists as typically described in terms of skills, competencies, qualities, or dispositions. Drawing on three agenda-setting texts from nineteenth-century history, biology, and sociology, the article argues that the “revolutions” these books sought to unleash were, among other things, revolts against inherited conceptions of scientific selfhood. They tried to redefine the scientific self in their respective fields of inquiry by advocating particular catalogs of virtues or character traits. These ideals of selfhood, their contested nature notwithstanding, translated into practice in so far as they influenced hiring and selection policies and found their way into educational systems. The project of reclaiming the scientific self as an important subject of study in the history of research ethics is not an antiquarian pursuit, but related to an ethical question faced by scientists today: How are their scientific selves being shaped by funding schemes, research evaluation protocols, and academic hiring policies?

## Introduction

The history of research ethics has often been reduced to the history of its codification. It has been reduced, more specifically, to codification in scientific codes of conduct such as the Nuremberg Code (1947) and the Declaration of Helsinki (1964). Textbooks on research ethics testify to the enduring power of this view when they trace the history of their field back to codification issued in response to medical experiments in Nazi Germany (e.g. Flynn and Goldsmith 2013: 9; Boddington 2012: 16; Fisher and Anushku 2008: 95; Wassenaar 2006: 61; Reiser 2002: 3; Bulger 2002: 117; Barnbaum and Byron 2001: 3). As long as research ethics is regarded as a realm of reflection different from scientific work itself—that is, different from data collection, hypothesis testing, statistical modelling, and the like—such an historical account is not implausible. Specifically, as long as historical inquiry is charged only with the task of providing historical antecedents to what is currently understood as research ethics—a key assumption of what the historian Herbert Butterfield (1931) called “Whig history”—there is little reason to question this textbook narrative.

The picture becomes considerably more complicated, however, as soon as we replace the closed-ended question “When has research ethics as we currently know it come into being?” with the open-ended question “How did scientists prior to the 1940s reflect on the ethical dimensions of their research?” Four recent trends in the history of medical ethics, as conveniently summarized by Robert Baker and Laurence McCullough (2009), illustrate just how much complexity this change of question adds to the history of research ethics. First, historians of medical ethics are expanding their subject matter to a variety of ethical discourses. Instead of focusing merely on language of rights and duties such as used in much of contemporary ethical reflection, they are increasingly also paying attention to older ethical discourses, including languages of honor, virtue, and “decorum” (Jonsen 2000; Baker 1999: 36–41; Haber 1991: 274–293). This is partly because, in the second place, historians of medical ethics are widening their range of source material. In a time when codes of conduct were still rare, ethical reflection on scientific conduct took place primarily in other genres, such as textbooks and professorial addresses on the scientific vocation (Baker 2013; Jonsen 2000: vi, xi). From this it follows, thirdly, that research ethics is much older than the term itself. Arguably, physicians in Renaissance Europe were already engaged in medical ethics when they reflected on the marks of a responsible doctor (Jonsen 2000: 43–56; Schleiner 1995). Finally, if research ethics amounts to reflection on the normative aspects of scientific conduct, there is no reason to maintain a traditional focus on “big issues” such as human experimentation (Schmidt and Frewer 2007). As Martin Pernick (2009: 16) argues, “value issues at stake in the nondramatic daily events” of scientific practice or health care are just as important a subject matter for historians of research ethics as the question “Should the doctor pull the plug?”

Perhaps the most important implication of this rewriting of the history of medical ethics is that research ethics is no longer regarded as distinct from science or medicine as such, but seen as permeating every aspect of the medical-scientific enterprise. The new approach thus challenges conventional distinctions between “scientific morality” (what scientists believe to be ethically responsible conduct) and “research ethics” (rules of conduct laid down in official ethical codes) (Baker 2013: 9–10) by recognizing ethical commitments in all normative assumptions as to how to engage responsibly in scientific research. This revisionism, however, also has a potential danger. If research ethics is becoming all-pervading, simply because all scientific activity is value-laden (Laudan 1986), then research ethics runs a risk of becoming indistinguishable from science itself. At some point, the word “ethics” ceases to have a distinct referent in historical reality.

So how can the history of research ethics be expanded beyond codification (the standard narrative) without losing all distinctiveness (the risk implied in the revisionist approach)? This article offers a way out of the dilemma by proposing a history of research ethics focused on the “scientific self,” that is, the role-specific identity of scientists as typically described in terms of skills, competences, qualities, or dispositions (Paul 2016b; Dumont 2008: 32–53; Daston and Galison 2007: 35–39, 198–205). This proposal has three advantages. First, by focusing on the scientific self and its defining qualities, it joins recent historians of medical ethics in challenging artificial distinctions between the history of science and the history of research ethics. Secondly, it expands the horizon beyond medicine so as to include other fields, from chemistry and philology to psychology and geography. Finally, and arguably most importantly, it allows for long-term histories, sensitive to variety in ethical idioms, while keeping a focus on the qualities believed to be conducive to responsible scientific conduct. The proposal thus allows for a wide scope, but also offers a focus.

Drawing on three agenda-setting texts from nineteenth-century history, biology, and sociology—Leopold Ranke’s *Zur Kritik neuerer Geschichtschreiber* (Critique of Modern Historians, 1824), Charles Darwin’s *On the Origin of Species* (1859), and Émile Durkheim’s *Les règles de la méthode sociologique* (The Rules of Sociological Method, 1895)—this article argues that the “scientific revolutions” these books sought to unleash were, among other things, revolts against inherited conceptions of scientific selfhood. All three authors tried to redefine the scientific self in their respective fields of inquiry by advocating particular catalogs of virtues or character traits. This article subsequently shows that these ideals of selfhood, their contested nature notwithstanding, translated into practice in so far as they influenced hiring and selection policies and found their way into educational systems. By way of conclusion, it is argued that the project of reclaiming the scientific self as an important subject of study in the history of research ethics is not an antiquarian pursuit, but related to an ethical question faced by scientists today: How are our selves being shaped by funding schemes, research evaluation protocols, and academic hiring policies?

## Three Classics

Darwin’s *Origin of Species*, to start with, is usually read as “one long argument” about natural selection as a primary mechanism for biological evolution. Yet at the same time, Darwin’s book is a treatise about the study of nature and, more specifically, the character traits that its author regarded as indispensable for a scientific naturalist. Judging by the opening paragraph, in which Darwin described the years of study that had gone into the book, patience and perseverance were prominent among these character traits: “I hope that I may be excused for entering on these personal details, as I give them to show that I have not been hasty in coming to a decision” (Darwin 1859: 1). If haste was detrimental to biological study as Darwin conceived of it, so were incautiousness, partiality, and “preconceived opinion.” Throughout the book, Darwin emphasized the importance of cautiousness, “dispassionate judgment,” and “flexibility of mind”—character traits that may be labelled “epistemic virtues” in so far as Darwin believed them to be indispensable for the epistemic project of understanding the nature of nature (Darwin 1859: 2, 6, 460, 482). More specifically, with clever rhetorical ploys, Darwin stylized himself as an author actually embodying these virtues (Levine 2011). The first person singular that emerges from Darwin’s pages is an “I” intent on overcoming “the blindness of preconceived opinion”—not by force or genius, but by cautious, patient, and open-minded inquiry (Darwin 1859: 483).

In the context of nineteenth-century England, this appeal to virtues conventionally associated with Isaac Newton, the undisputed hero of Victorian science (Higgitt 2007; Fara 2002; Yeo 1988), served two purposes at once. On the one hand, it was a defensive strategy, aimed at convincing skeptical readers that Darwin’s evolutionary theory strictly adhered to Newtonian principles of investigation (Bellon 2014: 223–224; Ruse 1999: 176). Unsurprisingly, this strategy worked only to an extent: it did not prevent critics from observing that Darwin had a proclivity for going where angels feared to tread. Although no one could object to open-mindedness as such—in Victorian England, “unflinching courage to declare the truths… how far soever removed from ordinary apprehension” even counted as a Newtonian virtue (Brougham 1858: 90)—the entomologist Thomas Vernon Wollaston was one among many who read the *Origin of Species* as a book full of “bold hypotheses and philosophical suggestions” (Wollaston 1860: 139), while the zoologist St. George Jackson Mivart, in response to *The Descent of Man* (1871), emphasized Darwin’s “extraordinarily active imagination” in developing bold hypotheses (Mivart 1871: 47; cf. Hull 1973: 135, 354).

More important, therefore, was Darwin’s second aim in emphasizing open-mindedness and independence of judgment as remedies to the “blindness of preconceived opinion.” These virtues not only befitted a man skeptical about received religious doctrine (Moore 1988), but also were of special importance to a scientist who preferred to work in the seclusion of his private estate, far away from universities and in cherished independence from colleagues or employers (White 2016). The virtue of open-mindedness in particular was central to a conception of scientific selfhood that privileged critical reasoning over dependence from peers, deference to senior colleagues, and respect for public sensitivities. No one grasped this better than Thomas Henry Huxley, nicknamed “Darwin’s bulldog,” whose extensive praise for Darwin was part of a campaign to establish a “new code of scientific conduct,” focused on rigorous inquiry more than on gentlemanly behavior (White 2003: 45). What interested Huxley most was not natural selection *per se*, but Darwin as “the incorporated ideal of a man of science,” characterized by virtues such as independence, courage, and an “almost passionate honesty by which all his thoughts and actions were irradiated” (Huxley 1882: 597). For Huxley, then, Darwin symbolized the emergence of a new scientific persona: the “professional scientist” for whom solving scientific questions was more important than fulfilling civic responsibilities—even if Darwin’s financial independence was out of reach for most emerging “scientists” (Mody 2016).

Similar concerns about the scientific self permeated Ranke’s “critique of modern historians,” which appeared at a time when Darwin was still at school and Ranke still a gymnasium teacher. The book is as rhetorically ingenious as Darwin’s and has arguably been as influential among historians as the *Origin of Species* has been among biologists. At first sight, it offers nothing more than a case-by-case examination of what Ranke calls the “trustworthiness” of early modern historians such as Francesco Guicciardini. Yet the results at which Ranke arrived had important implications for the historian’s self. When Ranke found the Florentine humanist guilty of “modification of facts” and “forgery of truth,” mostly caused by uncritical reliance on other authors, this implied that Guicciardini failed to live up to Ranke’s “modern” idea of what a historian should be (Ranke 1824: 24, 26). Even the most illustrious of early modern historians appeared no longer suitable as a model that modern historians could follow.

Like Darwin, therefore, Ranke proposed an alternative conception of selfhood, which he largely described in terms of virtues. “Trustworthiness,” for Ranke, required unflagging commitment to “naked truth without any adornment, thorough research of particulars… [and] no fabrication, not even in details” (Ranke 1824: 28). In highlighting these virtues, Ranke distanced himself not only from Renaissance historians, but also from older contemporaries such as G. W. F. Hegel, whose grand-scale philosophical history had little patience for “research of particulars,” and Friedrich Christoph Schlosser, whose moralizing habits obscured the “naked truth” as Ranke envisioned it. Initially, this revolutionary agenda was not particularly appreciated: colleagues such as Heinrich Leo ridiculed Ranke for his obsession with factual accuracy, while others saw Ranke as lacking “philosophical and religious seriousness” (Juhnke 2015: 54–55, 60; Baur 1998: 112–123). This criticism had little effect, though, as accuracy and precision were important qualities for a new type of historian that the mid-nineteenth century saw emerge: a source collector who spent long days in archival depositories, exploring the documentary record of the national past (Paul 2013). Thanks to a proliferating demand for source editions, in Germany as well as elsewhere, many of Ranke’s students found employment in projects that demanded philological virtues rather than philosophical vision or moral conviction (Weber 1984). Consequently, these students had little trouble turning Ranke into a model of “scientific history,” in comparison to which Leo’s Hegelian approach appeared as “pre-scientific.” In particular, Ranke was stylized into an epitome of “criticism,” “precision,” and “penetration,” as one pupil phrased it (Waitz 1867: 4), or into an embodiment of “objectivity,” as later generations would put it (Iggers 1962; Krill 1962; Paul 2017).

Objectivity was a key virtue for Durkheim, too, even though the rationalist leanings of the French sociologist lent this virtue a color different from Ranke’s. *Les règles de la méthode sociologique* is a passionate defense of research untainted by prejudice and fixed opinion. Cautioning against “the promptings of common sense,” “naivety,” “speculation,” and “dogmatism” (Durkheim 1982/1895: 31, 32, 37, 38), the book articulates a view of science that revolves around “mechanical objectivity” (Daston and Galison 2007: 121). For Durkheim, this implied, among other things, that a scientific sociologist has to “escape from the dominance of commonly held notions and to direct his attention to the facts” (Durkheim 1982/1895: 74).

This was more than a nineteenth-century update of Francis Bacon’s classic warning against “idols of the mind.” By contrasting “ideas” and “facts,” Durkheim engaged in no less than three polemics at once. At a most general level, he criticized a Cartesian-inspired type of philosophizing that he perceived as prioritizing generalization over patient collection of data. More specifically, he distanced himself from sociology as practiced by Herbert Spencer and Auguste Comte, both of whom he judged guilty of uncritically relying on philosophical generalizations for which no sociological evidence existed (Comte’s law of the three stages serves as an example: Durkheim 1982/1895: 64, 140). Closer to home, finally, Durkheim’s “explosive document” (Gane 1988: 19) was targeted against sociologists like Gabriel Tarde, who worked with a concept of individual agency that Durkheim reckoned among the unexamined ideas that scientists should abandon in favor of facts (Durkheim 1982/1895: 59). Durkheimian sociology, in short, was a rebellion against several inherited traditions of reflection on the social, characterized by firm rejection of all “vague impressions, prejudices, and passions” (Durkheim 1982/1895: 66).

At the same time, Durkheim was advocating a catalog of virtues that matched the kind of research that he organized around the *Année sociologique*—a journal that sought to enhance scientific standards through cooperation and mutual criticism between its contributors (Heilbron 2015: 82–91; Besnard 1983; Clark 1973: 181–186). Whereas Ranke’s philological virtues were most at home in a scientific culture that put a premium value on the collecting and editing of historical source material, Durkheim’s mechanical objectivity best fitted the collective research environment that the French sociologist created around the *Année sociologique*. This shows that the scientific self cannot be seen apart from its institutional contexts, on the one hand, and from the kind of research pursued in those contexts, on the other. The scientific self is being shaped by its environment, just as research questions and methods make their demands on it.

## Disciplining the Scientific Self

So here we have three scientific manifestos, from three different disciplines in three European countries, all of which called for a “revolution” in their respective fields of inquiry. Although, especially in Darwin’s case, these revolutions primarily challenged inherited scientific theories, the new interpretative models corresponded to a new conception of scientific selfhood. That is why all three authors located part of their revolutions within the self. At least to some extent, they sought to change science by changing the scientist—that is, by molding his (not yet her) dispositions or character traits. But how could this be done? How could virtues be acquired and vices be unlearned, especially if this challenged established views on the nature of a good scientist?

Ranke, Darwin, and Durkheim all dwelt at length on the efforts this required, meanwhile offering psychological explanations as to why their intended revolutions were long overdue and unlikely to win many hearts. Durkheim most explicitly pointed to the power of unconsciously acquired mental habits when he argued that no scientist is likely to overcome all prejudice: “The mind has such a natural disposition to fail to recognise” the power of preconceptions “that inevitably we will relapse into past errors” (Durkheim 1982/1895: 72). Darwin, too, elaborated on “the chief cause of our natural unwillingness” to accept such radical ideas as that evolution proceeds through natural selection: “[W]e are always slow in admitting any great change of which we do not see the intermediate steps” (Darwin 1859: 481). Ranke, finally, added that historians have reasons for altering historical truth: they care about the dramatic qualities of their narratives and seek to please their readers (Ranke 1824: 72, 76).

To justify their fights against these psychological obstacles, all three authors positioned themselves in long-term narratives of slow but steady scientific progress. Their rhetoric abounds with phrases like “as yet unexplained,” “much remains obscure,” and “still less do we know” (Darwin 1859: 6). Ignorance, indeed, is a key term in the *Origin of Species* (“nor do we know how ignorant we are”) (Darwin 1859: 466; see also Wallace 1995). Similar phrases—“we are virtually ignorant,” “we are only just beginning to perceive a few glimmers of light”—appear throughout Durkheim’s *Règles* (Durkheim 1982/1895: 38). Apparently, the scientific revolutions required scientists to subject their “prescientific selves” (Kaufman-Osborn 1987: 642) to strict scientific demands.

This led Durkheim to emphasize the importance of “rigorous discipline,” with all the Foucauldian connotations of that term: “Only sustained and special practice can prevent such shortcomings” (Durkheim 1982/1895: 72, 31). While Ranke provided such training in his *historische Übungen*—an informal seminar for advanced students that soon acquired the symbolic significance of a rite of initiation into the historians’ guild (Eskildsen 2007; Huttner 2001; Pandel 1994)—Durkheim’s educational facility was the *Année sociologique*, a journal (as we saw above) that socialized emerging sociologists into an ethos of objectivity by making them cooperate with each other, under Durkheim’s supervision. Darwin’s followers, too, agreed on the importance of “mental discipline,” although Huxley added that “enthusiasm for truth” is usually best kindled by exemplary figures who show in concrete detail what a scientific self looks like (Huxley 1874: 665). Unsurprisingly, Huxley used the occasion of the unveiling of a Darwin statue in the British Museum of Natural History to argue that Darwin, more than anybody else, embodied “the ideal according to which [future students of nature] must shape their lives” (Huxley 1885: 535).

## Moral Economies of Science

To what extent does such disciplining of scientific selves qualify as belonging to the realm of research ethics? From the perspective of the standard narrative summarized above, quarrels between competing scientific schools on the marks of a good scientist seem to be of only marginal relevance. However, if we follow recent historians of medical ethics in broadening research ethics so as to include the “moral economies of science,” defined as webs of emotionally charged values that cultivate “mental habits, methods of investigation, and even characters of a distinctive stamp” (Daston 1995: 23), then there are four reasons as to why the molding of scientific selves is an integral part of research ethics.

1. Although cautiousness, criticism, and objectivity are often classified as epistemic virtues, they were also heavily charged with moral meaning—to such an extent that the “epistemic” and the “moral” are often difficult to disentangle (Creyghton et al. 2016). For Ranke, Darwin, and Durkheim, cautiousness, criticism, and objectivity were virtues demarcating the difference between appropriate and inappropriate scientific conduct. Virtues were thus no supererogatory qualities, but preconditions for proper scientific work. The fact that not all scientists possessed those qualities to the same extent is no argument against them: virtue language specified the “ought,” not the “is” (Saarloos 2016; Paul 2012). Consequently, when nineteenth-century scientists debated the character traits characteristic of a “man of science,” they were discussing research ethical standards. In their eyes, skepticism towards established authority, detachment from political engagement, and indifference to monetary profit were markers of devotion to responsible scientific research.
2. The critical responses that Ranke’s, Darwin’s, and Durkheim’s manifestos elicited (Henz 2014: 127–133; Hull 1973; Gane 1988: 75–86) illustrate, among other things, that standards of virtue were not seldom contested. Even if few critics doubted the need for impartiality or criticism as such, scientists frequently found themselves disagreeing over the relative importance of these virtues (Paul 2015). Consequently, moral economies were not universally shared: they existed in the plural (Daston 1995: 3), as did the models that scientists invoked as embodiments of their ethics (Paul 2017). Such diversity of opinion about the marks of a scientific self does not distract, however, from their ethical significance. To the contrary, judging by the strong moral language sometimes used to criticize adherents of alternative models—“renegades who do not belong in the sanctuary of… science” (Lehmann 1895: 79)—it seems that the scientific self was subject to debate precisely because of its key position in a virtue-oriented type of research ethics.
3. The severity of the ethical issues at stake is apparent from the consequences that those involved were sometimes prepared to draw. When Huxley charged Richard Owen with not living up to Darwinian standards, he not only criticized his senior colleague in print, but also tried to block his election to the Royal Society Council on the ground that this would be a moral mistake (White 2003: 54). Likewise, Max Lehmann, one of Ranke’s students, not only drew public attention to what he perceived as the “unscientific” working manners of his intended Marburg successor, Albert Naudé, but also worked behind the scenes to prevent his appointment (Lehmann 1894: 129; Paul 2016a). Consequently, there was no lack of ethical boundary work, defined as deliberate attempts to exclude colleagues suspected of “unethical” conduct from the realm of science (Wainwright et al. 2006; Hesketh 2008).
4. For students, finally, ideals of virtue had tangible implications to the extent that they translated into pedagogical regimes. Part of academic socialization in nineteenth-century fashion consisted of ethical formation focused on the cultivation of character traits believed to be conducive to the practice of responsible science. Such cultivation of virtues not only took place in seminars and laboratories (Eskildsen 2007; Jardine 1992; Olesko 1991), but also ranked high among the aims of academic mentoring (Manteufel 2016) and, perhaps less obvious, team sports of the sort fervently played by students at Oxbridge colleges (Warwick 2003: 176–226). Although these practices obviously served more than research ethical goals, they were believed to contribute to ethical formation by fostering relevant character traits, not least including self-discipline and devotion to a common cause (Levine 2002; Anderson 2001). Research ethics was not usually the subject of special courses, but a matter of character formation to which informal academic socialization contributed at least as much as formal educational practices (Eskildsen 2015, 2016).

## Beyond the Nineteenth Century

If the analysis so far lends plausibility to the view that nineteenth-century scientists conceived of research ethics in terms of virtues or character traits, further argument is needed to sustain the claim that the scientific self is a suitable focus for a long-term history of research ethics. Specifically, two closely related issues need to be addressed: (1) Assuming that virtue language largely fell into disuse in the twentieth century, how do the virtues advocated by Ranke, Darwin, and Durkheim relate to the moral language prevalent in twentieth-century codes of conduct? Specifically, is there sufficient continuity to allow for long-term comparison? (2) Virtue is a relevant category as long as individuals can put their stamp on scientific research. But to what extent was that the case in the Cold War military-industrial-scientific complex, or is that the case in today’s globally entangled world of science? Does the scientific self still matter in an age of “science 3.0” (Miedema 2012)?

As for the first question, the assumption that virtue language has disappeared in the course of the twentieth century is in need of empirical testing. Whereas the generic term “virtue” has come to be imbued with Victorian connotations, this is not typically the case for dispositions conventionally classified as virtues, such as accuracy, precision, and objectivity. Whether scientists in, say, 1970 spoke less often about accuracy, precision, or objectivity than their predecessors in 1870 is a question still awaiting detailed historical inquiry. Additionally, it would be erroneous to think that codes of conduct had no space for virtue language. Codes of conduct were, and are, a heterogeneous genre, especially in that they often drew eclectically on several moral languages at once. Although it seems that early codes, such as those issued by the American Association for the Advancement of Science (1927) and the American Chemical Society (1947), used categories of virtue and honor more prominently than late twentieth-century ones, as late as 1980, the American Medical Association’s Code of Medical Ethics combined “deontology, decorum, and politic ethics,” as Albert Jonsen (2000: 117) has shown. Even the American Psychological Association, whose 1981 code clearly favored a language of “skills” and “competences,” could not resist invoking classic epistemic virtues, such as objectivity, carefulness, and accuracy (1981: 633, 634). Arguably, therefore, the story of virtue language in twentieth-century research ethics is more complicated than assumed in simple narratives of decline.

Secondly, even if detailed follow-up research would demonstrate an overall decline in virtue language, this would not imply that scientific selfhood became obsolete in an age of “science 3.0.” As Steven Shapin forcefully argues, the increase in scale and complexity characteristic of twentieth-century scientific research in and outside of academia did not produce an “elimination of the personal” of a sort proclaimed by conservative and progressive social theorists alike. To the contrary, it made increasing demands on individuals’ personal abilities to navigate complex professional environments. Although twentieth-century research managers might have smiled at the virtues advocated by Ranke, Darwin, or Durkheim, their job advertisements reveal how much they continued to care about “imagination, initiative, resourcefulness, energy, persistence, judgment, honesty, accuracy, dependability, loyalty and cooperativeness”—all of which are personal dispositions (Shapin 2008: 184). Likewise, whenever scientists in more recent decades tried to persuade venture capitalists to invest millions of dollars into research projects aimed at curing cancer, they made strategic use of time-honored repertoires of respectability and trustworthiness (Shapin 2008: 270). All this leads Shapin to conclude that the scientific self has anything but become obsolete, even if categories of virtue have frequently been replaced by skills and competences. “The closer you get to the heart of technoscience… the greater is the acknowledged role of the personal, the familiar, and even the charismatic” (Shapin 2008: 5).

A history of research ethics focused on the scientific self is therefore not a history of virtues alone, but a history of multiple discourses and practices on which scientists in various times and places drew in articulating, advocating, and implementing their research ethical standards. These discourses, in turn, were not confined to particular genres: they were used in different contexts, varying from informal *historische Übungen* to formalized codes of conduct. Yet what all these genres, discourses, and practices had in common is that they defined the scientific self and specified the expectations that scientists had to meet in order to be recognized as professionals. This, then, offers a suitable prism for a history of research ethics able to cover various periods, regions, scientific disciplines, and linguistic conventions. The history of research ethics proposed in this article is a history of demands placed upon the scientific self, in multiple languages, in several scientific genres, and in various research and educational practices.

## Conclusion

This implies, by way of conclusion, that a history of research ethics focusing on the scientific self *expands* the standard narrative, according to which research ethics emerged in response to World War II atrocities, into a longer and more intricate story about the demands that changing moral economies place upon scientists. Also, it *broadens* the subject matter of the history of research ethics by expanding its subject matter beyond ethical protocols. By putting the scientific self at center stage, it locates ethical reflection and formation right in the heart of scientific activity. Strategically, this encourages *cooperation* between historians of science and historians of research ethics. It invites the former to take seriously the ethical aspects of the scientific revolutions associated with the names of Ranke, Darwin, and Durkheim, just as it invites the latter to recognize that virtues such as advocated by these scientists were markers of a research ethics *avant la lettre*.

Finally, by way of coda, a study of moral demands made on scientists in times and places other than ours is not an antiquarian project. Examining how scientific selves were shaped and disciplined in earlier periods of history implicitly raises the question how scientific selfhood is molded in our days. What kind of ethical standards are implied in today’s academic reward structures? What sort of scientific conduct do competitive research funding schemes encourage (Mountz et al. 2015; Anderson et al. 2007)? How do social media and research evaluation protocols contribute to what is called a “quantified academic self” (Hammarfelt, De Rijcke, and Rushforth 2016)? Or what are the ethical subtexts of books like Donald Hall’s *The Academic Self: An Owner’s Manual* (2002)? Historical research of the kind proposed in this article holds up a mirror to present-day scientists, thereby encouraging them to examine the ethical implications inherent to current models of scientific selfhood.
